# Social Responsibility and Commitment to Children; Pediatric Nurses’ Experiences With Redeployment During the First Wave of COVID-19: A Qualitative Study

**DOI:** 10.1177/00469580241238419

**Published:** 2024-03-15

**Authors:** Nina Margrethe Kynø, Anette Winger, Edel Jannecke Svendsen, Tove Elisabeth Augustin Børsting

**Affiliations:** 1 Oslo Metropolitan University, Oslo, Norway; 2 Oslo University Hospital, Oslo, Norway; 3 Sunnaas Rehabilitation Hospital, Norway

**Keywords:** pediatric nursing, clinical competence, COVID-19, qualitative research

## Abstract

COVID-19 represented a challenge for health care worldwide and led to new tasks and a rethinking of resource use. It was necessary to establish capacity within hospitals and to reassign critical resources between hospitals. This study aimed to explore pediatric nurses’ experiences of redeployment, new tasks, and use of specialized competencies during the first wave of COVID-19. An exploratory design, involving qualitative individual interviews with 12 pediatric nurses was used. The analysis resulted in 3 main themes. Firstly, *a feeling of social responsibility* focused on how pediatric nurses felt committed to use their competencies during the crisis, whether they remained on the pediatric ward or were redeployed. Secondly, *fewer children to counterbalance the shortage of pediatric nurses* showed how redeployment was offset by fewer pediatric patients. Thirdly, *adapting pediatric nursing competencies to new tasks* described how the nurses adapted their skills to new tasks either in pediatric or adult wards. The results revealed that pediatric nurses had a social and ethical commitment to society in a crisis. They agreed to be redeployed and take on new tasks but were still concerned about the health and well-being of the children and their families, which led to a sense of ambivalence. They questioned whether their skills were being used appropriately in redeployment to adult wards. Fewer pediatric patients mitigated the workload of the remaining nurses. There is a risk of neglecting the needs of hospitalized children and their families during a pandemic. There was concern that “voluntary coercion” was a counterproductive strategy for reassignment.

**What do we already know about the topic?**
COVID-19 led to allocation and reallocation of staff in health care facilities. It was necessary to establish capacity within hospitals and to reassign critical resources between hospitals to achieve the required capacity. Nurses took on new tasks and responsibilities when relocated to other wards or in their own ward.
**How does your research contribute to the field?**
This study points out possible challenges to consider when reassigning pediatric nurses to adult wards, and how to deal with the challenges involved. Maintaining pediatric nursing competence for children and their families must be of great importance also in times of crisis.
**What are your research’s implications toward theory, practice or policy?**
An enhanced understanding of how a pandemic may affect pediatric nurses in a crisis can be useful for future planning of relocation and use of pediatric nursing competencies. Pediatric nurses can contribute their expertise to wards for adults during pandemics but should be prepared before being allocated to new wards.

## Introduction

Although COVID-19 affected countries differently, the pandemic represented a challenge for health care systems worldwide.^1^ Because of the rapidly growing number of seriously ill patients and the shortage of qualified health care personnel, COVID-19 led to the allocation and reallocation of staff in health care facilities.^2,3^ Rethinking the use of qualified personnel was also important in Norway, as it was necessary to establish capacity within hospitals and to reassign critical resources between hospitals to achieve the required capacity.^4^ In the early days of COVID-19, it was difficult to foresee its consequences and what impact it could have on different age groups. Health authorities^4^ were concerned about children’s general health, as scheduled consultations for diagnosis, assessment, treatment, and check-ups were postponed. Acute life-threatening treatment and long-term medical and emergency services continued with fewer changes, but children with symptoms that indicated treatment may have been overlooked,^4,5^ suggesting possible inadequate management of pediatric health care.

In general, due to more complex treatment trajectories and increasing survival rates for sick children, there is a need for pediatric nurses with specialized skills.^6^ A pediatric nurse is qualified to provide advanced nursing care to acute and critically ill and injured children from 0 to 18 years.^7^ In general, there is much discussion of nurses performing new tasks and being redeployed because of a persistent shortage specialized staff. It is expected that relocation and adaptation to new kinds of work will occur more frequently in the future, reinforced by a global nurse shortage.^8^

### Review of Literature

During COVID-19, nurses took on new tasks and responsibilities when relocated to other wards or in their own ward. Relocation to a different workplace with new duties is not necessarily a straightforward matter, since competencies can be difficult to apply at the same level in a new context. Cowan, Norman and Coopamah^9^ discuss nursing competence as the complex application of attitudes, values, knowledge, and skills situated in and closely connected to the context of the actual practice of nursing.

Hospitalized patients and their families experienced dramatic changes during COVID-19. Visiting restrictions imposed throughout the health care system had negative consequences for hospitalized children and their family members,^10,11^ and greater physical and emotional stress and workload for health care professionals.^11,12^ Some clinicians argued that the restrictions were necessary,^13^ while others found it difficult to compromise their work ethic by maintaining visiting restrictions and thus keeping families apart.^14^

Health care managers’ ability to redistribute staff and resources was hampered by a lack of information about COVID-19. The acute, unfamiliar, and insecure situation created general stress and moral distress among nurses in pediatric critical care. The uncertainty related to the pandemic made these nurses fear the virus for themselves and their family members.^15^ Pediatric nurses who were relocated to adult wards expressed concerns about safety, lack of preparation, emotional responses, and cooperation as a team.^16^ An enhanced understanding of how COVID-19 affected pediatric nurses in a crisis can be useful for future planning of relocation and use of pediatric nursing competencies.

The aim of this study is to explore pediatric nurses’ experience of redeployment and new tasks during the first wave of COVID-19. The study had 2 research questions:

(1) How did pediatric nurses experience being redeployed and performing new tasks during COVID-19?
(2) How did pediatric nurses feel about the use of their specialized competence during the first wave of COVID-19?

## Methods

This study is part of a larger study at Oslo Metropolitan University in Eastern Norway investigating the use of the competencies of specialized nurses during COVID-19.

The researchers’ positionality can be described through their teaching experience in a master’s degree program in pediatric nursing and in a postgraduate program in palliative care for children. They did not perform clinical work during the pandemic and thus did not work with any of the interviewees. Their fundamental understanding of pediatric nursing may have influenced their positionality in the research process.

However, by having 4 researchers working in different pairs, the influence of their pre-understanding on the research and the interviewees could be minimized.

### Design

We used an exploratory design involving individual qualitative interviews. Qualitative interviews are appropriate when the aim is to investigate participants’ perspectives on a phenomenon.^17^ Sample size should be guided by the study aim, sample specificity, the use of established theory, dialog quality and analysis strategy.^18^ The aim of this study was rather narrow as we sought pediatric nurses’ experiences during a specific period in history. Sample specificity is dense as the interviewees are a specific target group, namely pediatric nurses. However, there is no established theory concerning the aim of this study, which necessitated a higher number of interviewees. The dialog-based interviews were conducted by 2 experienced interviewers, which ensured the quality of the interviews. The sample of 12 pediatric nurses could be expected to provide enough information to be trustworthy, and to find patterns across the dataset, while also allowing for a focus on each individual interviewee.^18,19^

The interviews were audio recorded and transcribed verbatim by a research assistant. The transcripts were checked for accuracy by NMK and TEB.

### Sample

Invitations to participate in the study were sent by e-mail to 5 relevant leaders of pediatric wards in 2 university hospitals in eastern Norway. The leaders provided information about the study to the pediatric nurses, who then contacted the researcher if they were interested in being interviewed. Reminders were sent to the leaders once.

Participants were included if they had at least a postgraduate qualification in pediatric nursing and had been working in the pediatric ward when the first wave of COVID-19 began. Two experienced female interviewers conducted the 12 interviews together (NMK and TEB). The interviews were conducted in a dialog-based manner, following a predefined thematic interview guide (Supplemental File 1). The interviews were held in a quiet room in adjacent to the wards during June 2021.

The interviewees held high information power, as each interview gave rich information. All pediatric nurses who fulfilled the inclusion criteria and gave their consent to participate were interviewed.

### Ethical Considerations

All participants gave written informed consent to participate in the study. The study was approved by the Norwegian Centre for Research Data (NSD reference number: 566062) and the leaders of the wards. The leaders made the first contact with the participants. To ensure participant anonymity, pseudonyms are used in the results section.

### Analysis

After each interview, the interviewers (NMK and TEB) reflected together on the content and possible themes, making notes about content and how the interviews evolved.

The data analysis was inductive and inspired by Braun and Clarke’s^19,20^ 6 phases of thematic analysis. First, all researchers *familiarized* themselves with the data transcripts, read and re-read the data, and noted initial ideas (phase 1). The researchers *generated initial codes* in pairs (NMK/EJS and TEB/AW) (phase 2) and *searched for themes* (phase 3). The 2 groups met and *reviewed the initial themes* and discussed and compared the thematic map of the analysis (phase 4). Then, 1 member from each group (NMK and TEB) was responsible for the final *defining and naming of themes*. All 4 authors discussed the final themes until agreement was reached (phase 5). All authors participated in *producing a report of the analysis* as represented in the results section (phase 6).^19-21^

This study was reported in accordance with the consolidated criteria for reporting qualitative research (COREQ) checklist^22^ (Supplemental File 2). Rigor was obtained by having 4 analysts working in different pairs and all 4 together, discussing the findings until they reached agreement. They were pediatric nurses or specialists in nursing care of sick children.

## Results

### Sample Characteristics

All pediatric nurses who met the inclusion criteria were informed about the study, and all who agreed to participate were interviewed. The interviews provided rich data representing different experiences, perspectives, and stories. The interviewees were all women with at least a post-graduate qualification in pediatric nursing, aged between 29 and 61 years (mean 39.9 years). The nurses worked at 2 different university hospitals. Their clinical experience as pediatric nurses were between 2 and 23 years (mean 7.7 years). The interviews lasted from 20 to 43 min (mean 32 min) (Table 1).

**Table 1.** Demographic Data.

Participants	12
Gender	All women
Age	29-61 years (mean 39,9)
Clinical experience	2-23 years (mean 7.7)
Duration of the interviews	20-43 min (mean 32)

### Results to Answer the Research Question

The analysis resulted in 3 main themes. The first theme was *a feeling of social responsibility*, which focused on how the pediatric nurses felt committed to use their competencies and help out during a crisis, either to work on the pediatric ward or to be redeployed. The second theme was *fewer pediatric patients to counterbalance the shortage of pediatric nurses*, which showed how redeployment of pediatric nurses to other tasks was offset by a smaller number of children in the pediatric ward in this period. The third main theme was *adapting pediatric nursing competencies to new tasks.* In this third theme, the nurses described how they used their skills either in pediatric wards or in adult wards, and how they adapted to new tasks.

### A Feeling of Social Responsibility

During the first COVID-19 wave, half of the pediatric nurses interviewed remained on pediatric wards while the other half were reassigned to adult ICUs or enhanced monitoring wards (EMWs). One pediatric nurse described the reason for the redeployment to an adult ward:

*“Basically, we eased the workload in the ICU because ICU nurses were moved into the* [COVID-19] *cohorts.”* (Participant 4)

The redeployment to an adult ward was supposed to be voluntary, but the pediatric nurses did not feel that they had a choice. “It was voluntary coercion, I would say” (Participant 3).

Even though the pediatric nurses received little information about the transfer to adult wards, which entailed new tasks, unfamiliar environments, and new colleagues, they all felt committed to their social calling as health care professionals to play their part in this worldwide and national crisis. One nurse stated:

*“It’s so nice to see that there are so many who just want to contribute . . . in a crisis situation. There are so many people who just offer their assistance”* (Participant 7)

Although the nurses found it challenging to work with an adult population, they also felt that it was interesting and meaningful to use their competencies in the ICU. The nurse described it as “learning by doing” but also as “using the whole range” of her skills.

They occasionally felt lonely and missed their colleagues and the children in the wards, and the children’s parents. In general, the nurses had mixed feelings about the new assignments:

*“So it was a bit tough . . . It’s good to feel you’ve contributed”* (Participant 12)

Some nurses from different pediatric wards were reassigned to other pediatric wards, which involved both new and familiar work. Caring for pediatric patients with COVID-19 was not seen as distinct from taking care of other pediatric patients in isolation during infection. However, participant 9 said: *“The difference was that we had to wear [heavy] respiratory protection and goggles.”*

One nurse elaborated further on the general uncertainty about nursing care for acutely and critically ill children:

*“. . . we accepted children and relatives without knowing anything about how sick they were in the first place . . . Then our pediatric nursing skills are worth their weight in gold. It’s sort of much of the same during a pandemic or without a pandemic, you see. . .”* (Participant 8)

Although most nurses considered the reassignment to adult wards as an important contribution to strengthen the work in the COVID-19 cohorts, it did not come without costs. The nurses wanted to contribute during COVID-19 but were surprised that so many of the pediatric nurses were transferred from pediatric to adult wards. This made them concerned about the quality of care for the children in the pediatric wards. The nurses worried that children and their families lost their regular nurses, and that the children’s wards were deprived of much pediatric expertise.

The prominence given to the adult ICU patients concerned the pediatric nurses: “*Yes, I think they have let the kids down in a way”* (Participant 12). They questioned whether their specific nursing skills with children were so easy to replace when nurses without postgraduate pediatric qualifications took over their shifts:

“*We disagreed that it was okay to deploy so many . . . is it really that easy to replace us? It was a special situation, but the children also needed us during COVID-19*” (Participant 2)

### Fewer Pediatric Patients to Counterbalance the Shortage of Pediatric Nurses

When the nurses returned to their pediatric wards, it was hard work to catch up with unattended tasks that had been de-prioritized. Due to new tasks the pediatric nurses were concerned about insufficient parent support and information. Because of infection control the playrooms were closed, and the sick children therefore lost an important area for play, fun, and processing of bad clinical experiences. One nurse said:

*“Retrospectively, when we came back, there was a huge backlog that had been building up for a very long time. It was a huge workload.”* (Participant 1)

The lack of pediatric nursing skills in the pediatric wards was counterbalanced by the low patient occupancy in this period. However, although few children were hospitalized with COVID-19 infections, the restrictions impacted the child population admitted to the hospitals. Seasonal diagnoses such as respiratory syncytial virus infection, bronchiolitis and other respiratory illnesses were less frequent than in a normal virus season. The shortage of staff and insufficient pediatric nursing skills were solved because of the smaller numbers of pediatric patients.

*“. . . fewer children than usual were hospitalized during COVID-19 . . .the difference was what we were doing at work, I mean, with distancing and so on and infection control.”* (Participant 9)

In some pediatric wards, the nurses also treated COVID-19 pediatric patients. Because of the restrictions, only 1 parent was allowed to stay in strict isolation with the child in the pediatric ward. Nurses said that this affected both the child and the child’s family, as well as how the pediatric nurses worked with the families:

*“The only thing maybe is that they [parents] are more alone with the child, they may feel lonelier inside the infection control rooms. I think it’s difficult to understand how awkward it is to sit day in and day out in a room, when only [the staff] might stop by twice per shift.”* (Participant 6)

### Adapting Pediatric Nursing Competencies to New Tasks

Pediatric nursing education is based on a tradition of promoting children’s growth and development despite illness. Although both the reassigned nurses and those who remained in the pediatric wards agreed that the quality of care was ensured, they all felt that the parents and other relatives had suffered during COVID-19. This led to rethinking about how they worked and how they used their pediatric nursing competencies, both in the pediatric wards and in adult ICUs or EMWs.

Pediatric nurses in the ICU/EMW had conflicting experiences of how they were able to use their competencies, depending on their previous work experience. One said:

*“I have a lot of previous experience, but still there were things I’d never done that I had to think about a bit. . . It was incredibly demanding to be in that situation, and I was exhausted afterwards. But what I am left with is that I’ve learned a huge amount, and I feel I’ve actually used the whole range of myself and all my knowledge.”* (Participant 7)

The redeployed nurses were happy to receive training from the intensive care nurses and noted that it was easy to ask for help and explanations when needed. The pediatric nurses were warmly welcomed in the ICU/EMW by the staff, but they also felt skeptical about their contribution and whether pediatric nurses had sufficient knowledge and skill to treat adults. Some nurses received comments about their expertise:

*“In the beginning, it was like: who are you? . . .. But after a while, we got positive feedback . . . they noticed that we had extensive experience . . .”* (Participant 3).

One example is the pediatric nurses’ emphasis on collaboration with patients’ relatives:

*“I’m used to talking with relatives in a different way [than nurses working with adults]. We’re used to working more closely with relatives and they’re present all the time”* (Participant 3)

They also found that their pediatric skills were recognized as useful and enabled them to fulfill their mandate of taking on new tasks. The reassigned nurses reflected on the difference between ICU nurses and pediatric nurses and felt that pediatric nurses had more patience with the patients, were more used to families staying with the children all the time and had a lighter touch when caring for their patients. The nurses who remained in the pediatric wards were given new tasks due to COVID-19 and the associated restrictions.

*“We haven’t had as many others [support staff] with us as we usually have during, for example, clinical procedures, or the ‘bright spots’ for the children. Yes, as I said, I thought they [the parents] might be very alone . . .”* (Participant 6)

Parents and children were isolated in patient rooms and were not allowed to leave. Not being able to take turns with the other parent as often as they wished was difficult. For mothers of newborns, this was particularly difficult because of breastfeeding, which meant that the mothers had to stay with their infant continuously.

Although the pediatric nurses questioned whether their competence was used in the right way in the ICU, they received recognition of their skills and contributions from colleagues in the adult ICU. Despite the demanding situation, the interviewed nurses stated that high quality care was ensured. The children’s ward seemed to be an unfamiliar environment without children playing, with no clowns and closed playrooms. The nurses were also worried about the parents and other relatives being stuck in the ward alone, with fewer possibilities to take turns with the other parent or another family member.

## Discussion

*Three main themes were identified in this study.* How the pediatric nurses experienced being redeployed and performing new tasks is described in theme 1, *a feeling of social responsibility*. How they felt about the use of their specialized competence is described in theme 2, *fewer pediatric patients to counterbalance the shortage of pediatric nurses* and theme 3, *adapting pediatric nursing competencies to new tasks.*

Nurses in this study described their experiences of being redeployed as both challenging and meaningful in taking social responsibility. However, as in other studies, the redeployed pediatric nurses found the sudden decision about redeployment difficult.^23^ The nurses had expected to work with COVID-19 patients, but most of them were redeployed to ICUs or EMWs, thereby releasing ICU nurses to work with COVID-19 patients. The pediatric nurses were proud of their contributions during COVID-19. This feeling of being useful might explain why they felt the repositioning to be interesting and meaningful. This feeling of pride is in line with previous research.^24^ When a crisis such as COVID-19 occurs, nurses have shown a social and ethical commitment to society, and adapt and accept challenges when working in times of crisis.^25^ Their adaptive ability may explain why the nurses in this study expressed social responsibility and agreed to redeployment with new tasks, despite feeling that being moved to other wards was not entirely voluntary. They expressed a feeling of ambivalence about leaving the pediatric wards being concerned about the health and well-being of the children and their families while making an important contribution in adult wards.

Redeploying pediatric nurses to care for adult COVID-19 patients does not seem to be exceptional,^3,16,26^ but it is a cause for concern if pediatric nurses in general are seen as an additional source of labor that must leave children’s wards in times of crisis. The pediatric nurses in this study felt that they let the children down, and they questioned whether their competencies were easy to replace. One may ask whether more pediatric nurses should have stayed in the children’s ward to maintain pediatric expertise and to ensure specialized care for acutely sick children. The rationale for the social responsibility that the nurses expressed can be found in the International Code of Ethics for Nurses (3.7), which requires nurses to prepare for and respond to health care needs such as pandemics. Individual nurses and health care leaders and organizations have a shared responsibility to use resources adequately.^27^

Another central finding in this study was that the pediatric nurses found that although they were fewer in number in the pediatric ward, this was counterbalanced by fewer children hospitalized. Nurses remaining in the pediatric wards provided care to both children and parents of unknown infection status or patients already diagnosed with COVID-19. Since their workload was lower than usual, there was less need for specialized pediatric competence due to the low number of children admitted at that time. In line with the study by Veerapen and Mckeown,^25^ pediatric nurses in this study were considered adaptable, flexible, and with core competencies that fulfilled the nursing requirements during the first wave of COVID-19. Our findings show that pediatric nurses are devoted to their work and that their expertise is important to provide specialized, high quality care to children and their families.

Redeployment of pediatric nurses to adult care came at the expense of the children and their parents in the pediatric wards. Historically, children’s health care has been less prioritized than that of adults.^28^ This also seemed to be the case during COVID-19, probably reinforced by reports about how children were less affected by the virus,^29,30^ and it became less valid to advocate for children’s needs when adults were dying.^31^ However, hospitalized children and their parents clearly suffered during the pandemic,^10,32^ and the pediatric nurses were all concerned about the families, and the strain for parents and children of remaining isolated for too long. They were also concerned about potential parental emotional and physical fatigue when only 1 parent was allowed to stay with the child. These statements reflect the part of the pediatric nurse’s competence that concerns values and attitudes. The disruption of these values and attitudes in addition to the general strain of the COVID-19 situation seemed to cause stress in these nurses. Working during excessive periods of stress may cause burnout and high rates of sick leave.^33^

Work in an unfamiliar field involves uncertainty about how to deliver what is expected and taking on a professional role outside one’s scope of expertise, which may compromise professional integrity and lead to moral distress.^15^ Initially, neither the nurses in the adult wards nor the pediatric nurses themselves knew how to use pediatric competencies in the new situation. The pediatric nurses in our study did not consider themselves prepared to care for adult patients, which has been shown to be challenging.^16^ One nurse described “learning by doing” and “using the whole range of her skills.” The interviews reveal not only how the nurses used a range of skills and knowledge, but also drew on their values and attitudes in their desire to provide high quality care and to learn in a new context.^9^ Pediatric competence was expanded and developed through training and asking questions. The pediatric nurses were also able to maintain their professional integrity as they joined other nurses and health professionals in solving care challenges in an overloaded health care system.

Working in a new environment was one of the most challenging factors for reassigned nurses during COVID-19.^24^ Our participants described how they missed their colleagues and occasionally felt lonely. This may have had a negative impact on their experience, as connectedness with colleagues, sharing stories, and mutually supporting each other are seen as positive factors for well-being in a new workplace.^15,16^ Since competence is attached to context (Cowan et al^9^), many pediatric nurses might have felt less competent in the new setting, which also affected their well-being.

Society can hardly rely on nurses’ social commitment to stay in their jobs, and it has become increasingly difficult to recruit and retain nurses since COVID-19.^8^ The ICN Policy Brief Nurse Shortage and Retention Report (2021) points out that the problem post-COVID-19 is that nurses are leaving the profession. One way to ensure retention could be to involve nurses in decisions about the use of their competence. Taking a broader view of competence that links it closely to a nurse’s values and context suggests that a nurse’s performance can be negatively affected when applied to a new context, especially if it is not voluntary. Highly educated employees with specialized, independent roles and responsibilities should take it for granted that they are consulted. If the worst predictions come true, there will be a long-term understaffing crisis and competition for nursing competence, and appealing to social responsibility will be insufficient to motivate nurses to stay in the profession. Experience from COVID-19 shows how our society will also need skilled health care professionals for possible crises in the future.

## Strengths and Limitations

One strength of this study was that the interviews were conducted by 2 interviewers. Further, the authors’ participation throughout the analysis ensured that various interpretations emerged and were discussed and agreed upon for the final themes. In a global perspective, the transferability of the results of this study concerns the use of special competencies of pediatric nurses, redeployment and the performance of new tasks in pandemic and crisis management. One limitation is the convenience sample as a strategy, since there may be a risk that the pediatric nurses participating in the study were particularly affected by COVID-19. The interviews were conducted 1 year after the first wave, which may have impacted the participants’ memory, but may also have led to greater reflection on their experiences during the period.

## Implications for Practice

This study may provide knowledge to improve pandemic management. First of all, a decision on how to use pediatric nurses’ competencies in the best way should be taken with a high degree of involvement of the nurses. Pediatric nurses can contribute their expertise to wards for adults during pandemics but should be better prepared before being allocated to new wards. However, there is also a need for their expertise in children’s wards as children need specialist nurses to take care of their development and special needs. This study points out possible challenges to consider when reassigning pediatric nurses to adult wards, and how to deal with the challenges involved. Maintaining the specific pediatric nursing competence for children and their families must be of great importance also in times of crisis.

## Conclusion

Our findings suggest that pediatric nurses have a social and ethical commitment to society when a crisis such as the COVID-19 pandemic occurs. They accepted being redeployed and receiving new tasks. At the same time, they were still concerned about the health and well-being of the hospitalized children and their families. This led to a sense of ambivalence when they had to leave the pediatric wards to assist in the ICU or EMW. Nurses who remain in pediatric wards caring for patients with COVID-19 and with unknown infection status need pediatric nursing skills in order to meet the special needs of the children and their family members.

Further, we found that the pediatric nurses were devoted to their work and took good care of the children and their families. They questioned whether their competence was used appropriately when they were reassigned to adult wards. Low occupancy in the pediatric wards partly prevented a high workload for the remaining nurses. An important learning point from this study is the risk of neglecting the needs of hospitalized children and their families during pandemics. There was also a concern that voluntary coercion was a counterproductive strategy for redeployment.

## Supplemental Material

sj-docx-1-inq-10.1177_00469580241238419 – Supplemental material for Social Responsibility and Commitment to Children; Pediatric Nurses’ Experiences With Redeployment During the First Wave of COVID-19: A Qualitative Study Supplemental material, sj-docx-1-inq-10.1177_00469580241238419 for Social Responsibility and Commitment to Children; Pediatric Nurses’ Experiences With Redeployment During the First Wave of COVID-19: A Qualitative Study by Nina Margrethe Kynø, Anette Winger, Edel Jannecke Svendsen and Tove Elisabeth Augustin Børsting in INQUIRY: The Journal of Health Care Organization, Provision, and Financing
