# Supplementary material for: Social Responsibility and Commitment to Children; Pediatric Nurses’ Experiences With Redeployment During the First Wave of COVID-19: A Qualitative Study
Source: Inquiry. 2024 Mar 15;61:00469580241238419. doi: 10.1177/00469580241238419 (PMC10943705; doi:10.1177/00469580241238419)
Supplement: sj-docx-1-inq-10.1177_00469580241238419 – Supplemental material for Social Responsibility and Commitment to Children; Pediatric Nurses’ Experiences With Redeployment During the First Wave of COVID-19: A Qualitative Study [file sj-docx-1-inq-10.1177_00469580241238419.docx]

Table 1. Interview guide

| **Responsibilities** | - Please tell how you experienced your work situation during the pandemic - Please tell us what tasks and responsibilities you had during the pandemic that you do not normally have? - If you were given other tasks than normal; how did you make use of your skills? Please give some examples. (use/not use of competence) - Have you had other colleagues than usual during the pandemic? - Please tell us about your experiences of working together during the pandemic |
| --- | --- |
| **Patient and relatives** | - In your opinion, how have the patients been taken care of during this period? - Please tell about a situation where a patient was well taken care of and a situation where a patient was not so well taken care of - What experiences do you have with caring for relatives during the pandemic? (Information, participation and support) - What role did relatives play during the pandemic? |
| **Concluding questions** | - Thinking back; can you highlight something that was particularly nice, or something that was particularly hard? - Thinking back; is there anything you wished had been done differently? - Finally, Is there anything you want to say more about, something you have not told us? |
